# Supplementary material for: Identification of photoperiod-induced specific miRNAs in the adrenal glands of Sunite sheep (Ovis aries)
Source: Front Vet Sci. 2022 Jul 22;9:888207. doi: 10.3389/fvets.2022.888207 (PMC9354845; doi:10.3389/fvets.2022.888207)
Supplement: Supplementary file 1 [file Data_Sheet_1.ZIP › Supplementary file/miRDeep2 file.zip!/miRDeep2 file/Know_Structure/SP42A1/oar-mir-376d.pdf]

miRBase precursor : oar-mir-376d
Total read count : 143
oar-miR-376d read count : 138
remaining reads : 5

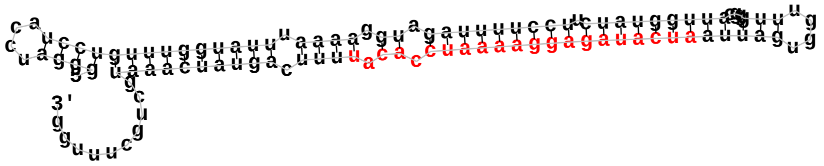

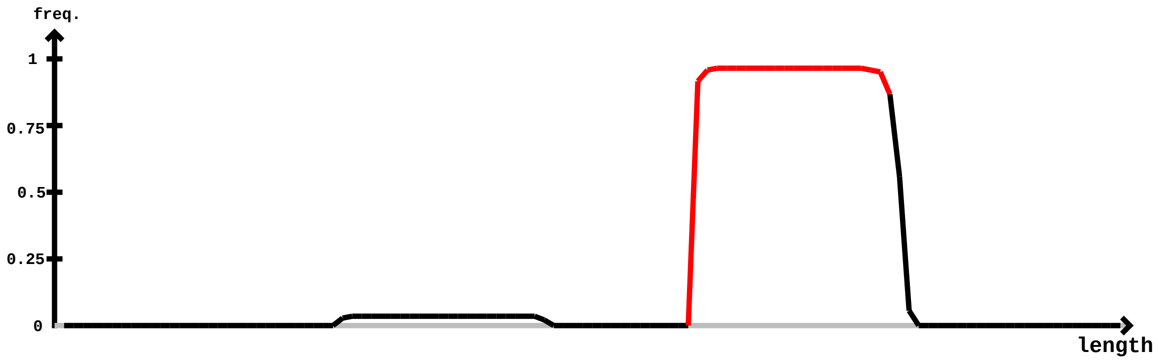


```
                                                                    oar-miR-376d
5'- gggauccauccuguuugguauuuaaaagguagauuuuccuucuaugguuacggguuuggugauuaaucauagaggaaaauccacauuucaguaucaaaugcugcuuugg   -3'    exp
    ((((....))))((((((((((.((((.((.(((((((((.(((((((((...(((....))))))))))))))))))))))))).)).)))).))))))))))..........   reads      mm         sample
    ............................guagauuuuccuucuaugguu..............................................................   2          0          seq
    ............................guagauuuuccuucuaugguua.............................................................   2          0          seq
    .............................uagauuuuccuucuaugguua.............................................................   1          0          seq
    ..................................................................aucauagaggaaaaucca.............................   1          0          seq
    ..................................................................aucauagaggaaaauccac............................   1          0          seq
    ..................................................................aucauagaggaaaauccaca...........................   12         0          seq
    ..................................................................aucauagaggaaaauccacau..........................   43         0          seq
    ..................................................................aucauagaggaaaauccacauu.........................   71         0          seq
    ..................................................................aucauagaggaaaauccacauuu........................   3          0          seq
    ...................................................................ucauagaggaaaauccacau..........................   1          0          seq
    ...................................................................ucauagaggaaaauccacauu.........................   1          0          seq
    ...................................................................ucauagaggaaaauccacauuu........................   4          0          seq
    ....................................................................cauagaggaaaauccacauuu........................   1          0          seq
```
